# Clinical and corneal microbial profile of infectious keratitis in a high HIV prevalence setting in rural South Africa

**DOI:** 10.1007/s10096-016-2677-x

**Published:** 2016-05-28

**Authors:** E. Schaftenaar, R. P. H. Peters, G. S. Baarsma, C. Meenken, N. S. Khosa, S. Getu, J. A. McIntyre, A. D. M. E. Osterhaus, G. M. G. M. Verjans

**Affiliations:** 1 Department of Viroscience (room Ee1720a), Erasmus Medical Center, ’s-Gravendijkwal 230, 3015 CE Rotterdam, The Netherlands; 2 Anova Health Institute, 12 Sherborne Road, Parktown, Johannesburg, South Africa; 3 Anova Health Institute, 21 Peace street, P.O. Box 2243, 0850 Tzaneen, South Africa; 4 Department of Medical Microbiology, University of Pretoria, cnr Lynnwood Road and Roper Street, Hatfield, Pretoria South Africa; 5 Rotterdam Ophthalmic Institute, Schiedamse Vest 160, 3011 BH Rotterdam, The Netherlands; 6 Department of Ophthalmology, VU University Medical Center, De Boelelaan 1118, 1081 HZ Amsterdam, The Netherlands; 7 School of Public Health & Family Medicine, University of Cape Town, Observatory, 7925, Cape Town, South Africa; 8 Research Center for Emerging Infections and Zoonoses, University of Veterinary Medicine, Medicine Bünteweg 17, 30559 Hannover, Germany

## Abstract

The purpose of this investigation was to determine the clinical and corneal microbial profile of infectious keratitis in a high human immunodeficiency virus (HIV) prevalence setting in rural South Africa. Data in this cross-sectional study were collected from patients presenting with symptoms of infectious keratitis (*n* = 46) at the ophthalmology outpatient department of three hospitals in rural South Africa. Corneal swabs were tested for herpes simplex virus type 1 (HSV-1) and 2 (HSV-2), varicella zoster virus (VZV) and adenovirus DNA by real-time polymerase chain reaction (PCR) and for bacteria and fungi by culture. Based on clinical history, disease characteristics and laboratory results, 29 (63 %) patients were diagnosed as viral keratitis, including 14 (48 %) viral keratitis cases complicated by bacterial superinfection, and 17 (37 %) as bacterial keratitis. VZV and HSV-1 DNA was detected in 11 (24 %) and 5 (11 %) corneal swabs, respectively. Among clinically defined viral keratitis cases, a negative viral swab was predominantly (93 %) observed in cases with subepithelial inflammation and was significantly associated with an increased duration of symptoms (*p* = 0.003). The majority of bacteria cultured were Gram-positive (24/35), including *Staphylococcus epidermidis* and *S. aureus*. Viral aetiology was significantly associated with a history of herpes zoster ophthalmicus (*p* < 0.001) and a trend was observed between viral aetiology and HIV infection (*p* = 0.06). Twenty-one (47 %) keratitis cases were complicated by anterior uveitis, of which 18 (86 %) were HIV-infected cases with viral keratitis. The data implicate a high prevalence of herpetic keratitis, in part complicated by bacterial superinfection and/or uveitis, in HIV-infected individuals presenting with infectious keratitis in rural South Africa.

## Introduction

Infectious keratitis is a major cause of ocular morbidity worldwide and the most common cause of unilateral corneal blindness in low-resource settings [1]. The estimated population incidence of infectious keratitis in these settings is up to 800 per 100,000/year, which is about 70 times higher than in high-resource settings [1]. Early diagnosis is essential, as visual outcome depends on the prompt initiation of targeted antimicrobial treatment [2, 3]. The spectrum of keratitis-associated pathogens is diverse and includes viruses, bacteria, fungi and protozoa. Moreover, the clinical picture and aetiology of infectious keratitis varies geographically, as it is subject to both environmental and host factors [1, 2].

Viruses, in particular human alpha herpesviruses, are well known in Western countries for causing recurrent and devastating keratitis, but data from sub-Saharan African countries are scarce and solely based on clinical diagnosis [4–6]. The high human immunodeficiency virus (HIV) prevalence in this region may play an important role in the distribution of keratitis-associated pathogens, as HIV-infected individuals are at higher risk for viral keratitis, particularly varicella zoster virus (VZV) keratitis [7]. However, the management of infectious keratitis in this region predominantly involves (presumptive) antibiotic and/or anti-fungal treatment, which may lead to inappropriate treatment of patients with viral keratitis, thereby increasing the risk of visual disability [8]. Elucidation of both the aetiology of infectious keratitis, in particular the potential role of viruses, and the associated clinical picture in a high HIV prevalence setting such as rural South Africa is of paramount importance to improve diagnosis and clinical management aimed to prevent visual impairment and blindness.

The aim of the current study was to determine the clinical and corneal microbial profile of infectious keratitis in a high HIV prevalence setting in rural South Africa.

## Materials and methods

### Study population and setting

This cross-sectional study was conducted at the ophthalmology outpatient department of three hospitals in rural South Africa (Mopani District) between September 2013 and May 2015. Criteria for participation were adult age (≥18 years old), no recent history of ocular surgery or trauma, willingness to test for HIV and a clinical diagnosis of keratitis based on slit-lamp examination: inflammation of the cornea with or without the presence of a corneal epithelial defect [2, 3, 9]. Contact lens wearers were excluded. Infectious keratitis was classified as viral, bacterial, fungal and/or protozoan on the basis of clinical history (e.g. history of unilateral painful skin rash in the dermatomal distribution of the trigeminal nerve as a sign of VZV infection), disease characteristics (e.g. typical herpetic corneal dendrites, disciform keratitis or an epithelial defect associated with a larger infiltrate as a sign of bacterial keratitis), laboratory results and response to initiated treatment according to current diagnostic criteria [3, 10, 11]. Infectious keratitis patients presenting with uveitis were defined as keratouveitis. Treatment of infectious keratitis was initiated according to standard treatment guidelines for hospitals from the National Department of Health of South Africa [12]. Ethical clearance for this study was obtained from the Human Research Ethics Committee (University of the Witwatersrand, Johannesburg, South Africa; project ID: M130201). Written informed consent was obtained from all participants.

### Ophthalmic examination

Demographic and clinical data were collected using a questionnaire and full ophthalmic examination was performed, including visual acuity using an ‘Illiterate E’ Snellen chart at a distance of 6 m, slit-lamp examination, intraocular pressure using the Icare TA01i (Icare Finland Oy, Helsinki, Finland) and dilated indirect funduscopy. Visual acuity after treatment was also determined at routine clinical follow-up visits. Visual impairment was defined according to the International Classification of Diseases on the basis of the individual’s visual acuity [13]. Counselling and HIV testing following routine practice were performed for all participants who reported to be HIV-uninfected or were unaware of their HIV status. Diagnostic CD4 counts were determined in all HIV-infected participants.

### Laboratory analyses

Corneal samples were collected from the affected eye under topical anaesthesia using a corneal swab and an eyelid spreader to prevent contamination from the eyelids. Due to the lack of ophthalmological care in this region, we chose to perform corneal swabbing instead of corneal scraping, as complications due to corneal swabbing are less likely to occur. All samples were collected by the same investigator (ES). First, a corneal swab for virus detection was obtained in 5 mL collection medium (Puritan Diagnostics, Guilford, CT, USA). Viral swabs were examined for herpes simplex virus type 1 (HSV-1), HSV type 2 (HSV-2), VZV and adenovirus DNA by real-time polymerase chain reaction (PCR) using virus-specific primer/probe combinations at the diagnostic laboratory of the Department of Viroscience of Erasmus Medical Center (Rotterdam, the Netherlands), as described elsewhere [14]. The sensitivity of the virus-specific real-time PCR assays, as defined by the 95 % hit rate on the electron microscopy counted virus stocks, was about 100 virus genome-equivalent copies/mL. A second swab (Transystem™) for bacterial and fungal culture (Copan Diagnostics Inc., Murrieta, CA, USA) was obtained from the same eye. Microbial examination, including Gram stain microscopy and culture, was performed on swabs for bacteria and fungi at the Lancet Laboratory according to standard diagnostic procedures (Tzaneen, South Africa).

### Statistical analysis

Data were double-entered into Epi Info version 3.5.4 [Centers for Disease Control and Prevention (CDC), Atlanta, GA, USA] and analysed using IBM SPSS Statistics version 22 (IBM, New York City, NY, USA). Descriptions of the study population and clinical and laboratory findings were performed using number with proportion and median with interquartile range (IQR) and stratified to HIV status. Demographic, clinical and laboratory factors were compared between different aetiologies of keratitis to identify factors associated with infectious keratitis. Comparison was done by Chi-squared tests with Fisher’s exact test if appropriate for categorical variables and the Mann–Whitney test for continuous variables. Data are presented as an odds ratio (OR) with 95 % confidence interval (CI) or as a median with IQR.

## Results

### Demographics and clinical presentation

We recruited 46 patients clinically diagnosed with infectious keratitis, consisting of 29 (63 %) women and 17 (37 %) men, with a median age of 41 (IQR 31–59) years (Table 1). Twenty-eight (61 %) participants were HIV-infected, of which 6 (21 %) were tested reactive to HIV for the first time and 13 (46 %) were on antiretroviral therapy (ART). The median CD4 count at enrolment was 226 (IQR 156–329) CD4 T-cells/mm^3^ for those on ART and 299 (IQR 160–396) CD4 T-cells/mm^3^ for ART-naïve participants.

**Table 1:** Characteristics of infectious keratitis patients enrolled in this study

	HIV infected (*n* = 28)	HIV uninfected (*n* = 18)	Crude odds ratio (95 % CI)	*p*-Value
Gender				
Female	22 (76)	7 (24)	5.8 (1.6–21.3)	0.01
Male	6 (35)	11 (65)		
Age in years	38 (31–45)	52 (27–72)	na	0.19
Ethnicity				
Sotho	19 (66)	10 (34)	1.7 (0.5–5.7)	0.53
Shangaan	9 (53)	8 (47)		
Low educational status	10 (36)	9 (50)	0.6 (0.2–1.9)	0.37
Low financial income	22 (79)	14 (78)	1.0 (0.3–4.4)	1.00
CD4 cell count in cells/mm^3^	254 (162–353)	na	na	na
Days between onset of eye complaints and presentation	18 (11–49)	23 (9–38)	na	0.93
Referred from primary healthcare facility	18 (64)	8 (44)	2.3 (0.7–7.5)	0.23
Use of topical antibiotics prior to enrolment	10 (36)	4 (22)	1.9 (0.5–7.5)	0.51
Diagnosis (clinical and laboratory data combined)				
Viral keratitis (*n* = 15)	10 (67)	5 (33)	3.7 (1.1–13.3)^a^	0.06
Viral and bacterial keratitis (*n* = 14)	11 (79)	3 (21)		
Bacterial keratitis (*n* = 17)	7 (41)	10 (59)		

Data are shown as number (%) or median (interquartile range)

*CI* confidence interval; *p-Value* Pearson Chi-square or Mann–Whitney U test; *na* not applicable; *HIV* human immunodeficiency virus

^a^Crude odds ratio and *p*-values were calculated for viral keratitis (including viral and bacterial keratitis) vs. bacterial keratitis

Reduced vision was the most common complaint reported at enrolment (100 %), followed by eye pain (96 %), tearing (72 %) and photophobia (65 %). The median duration of symptoms was 19 (IQR 11–38) days. Twenty-six (57 %) patients had been referred from a primary healthcare (PHC) facility. Fourteen (30 %) patients reported the use of topical antibiotic eye ointment (Chloramphenicol) prior to inclusion; unfortunately, only three patients (21 %) used the ointment adequately. At ophthalmic examination, an epithelial defect was the most commonly observed clinical characteristic (65 %), followed by signs of anterior chamber inflammation (52 %). Corneal infiltration was the most common clinical characteristic associated with an epithelial defect (33 %), followed by corneal dendrites (27 %), punctate epithelial keratitis (20 %) and corneal ulceration (20 %). In cases of subepithelial inflammation, stromal keratitis was the most often observed clinical characteristic (56 %), followed by subepithelial infiltration (31 %) and endothelial inflammation (13 %). An intraocular pressure >21 mmHg was observed in 9 of 43 (21 %) patients.

### Microbial laboratory analyses on corneal swabs

Viral DNA was detected from corneal swabs in 16 of 45 (36 %) patients; one viral corneal swab was unavailable for viral diagnostics. Whereas HSV-2 and adenovirus DNA was undetectable by PCR, 11 (24 %) and 5 (11 %) swabs were positive for VZV [median PCR cycle threshold (Ct) value of 37.0 (IQR 32.2–38.9)] and HSV-1 [median PCR Ct value of 33.0 (IQR 26.6–36.7)] DNA, respectively (Table 2). Bacteria were cultured from the corneal swabs of 29 (63 %) patients, mainly Gram-positive bacteria (69 %) with *Staphylococcus epidermidis* as the most common detected bacterium (36 %), followed by *S. aureus* (14 %) and *S. capitis* (9 %). Based on clinical history, disease characteristics, laboratory results and response to initiated treatment (i.e. antibiotics or antiviral), 29 (63 %) patients were diagnosed as viral keratitis, including 14 (48 %) viral keratitis cases complicated by bacterial superinfection and 17 (37 %) as bacterial keratitis (Table 2).

**Table 2:** Aetiology of infectious keratitis defined by clinical and laboratory diagnostic methods

		HSV-1 PCR^POS^	VZV PCR^POS^	Microbial culture positive
HIV-infected patients (*n* = 28)				
Viral keratitis (*n* = 10)				
Laboratory-confirmed diagnosis (*n* = 4)	*n* = 2	*n* = 2		No bacterium cultured
Clinical diagnosis only (*n* = 6)	None	None		No bacterium cultured
Viral and bacterial keratitis (*n* = 11)				
Laboratory-confirmed diagnosis (*n* = 8)	*n* = 1	*n* = 7		*Staphylococcus epidermidis* (*n* = 3) *Staphylococcus aureus* (*n* = 2^a^) *Bacillus* species (*n* = 1) *Stenotrophomonas maltophilia* (*n* = 1) *Staphylococcus epidermidis* and *Escherichia coli* (*n* = 1)
Clinical diagnosis only (*n* = 3)	None	None		*Staphylococcus capitis* (*n* = 1) *Staphylococcus epidermidis* (*n* = 1) *Enterobacter cloacae* (*n* = 1)
Bacterial keratitis (*n* = 7)^b^				
Laboratory-confirmed diagnosis (*n* = 7)	None	None		*Staphylococcus epidermidis* (*n* = 2) *Pseudomonas aeruginosa* (*n* = 2) *Staphylococcus capitis* (*n* = 1) *Staphylococcus aureus* and *Streptococcus viridans* (*n* = 1) *Corynebacterium*, *Enterococcus faecalis*, *Escherichia coli* and *Candida albicans* (*n* = 1)
Clinical diagnosis only (*n* = 0)	None	None		No bacterium cultured
HIV-uninfected patients (*n* = 18)				
Viral keratitis (*n* = 5)				
Laboratory-confirmed diagnosis (*n* = 2)	*n* = 2	None		No bacterium cultured
Clinical diagnosis only (*n* = 3)	None	None		No bacterium cultured
Viral and bacterial keratitis (*n* = 3)				
Laboratory-confirmed diagnosis (*n* = 1)	None	*n* = 1		*Staphylococcus aureus* and *Proteus mirabilis* (*n* = 1)
Clinical diagnosis only (*n* = 2)	None	None		*Staphylococcus epidermidis* (*n* = 1) *Staphylococcus capitis* and *Acinetobacter haemolyticus* (*n* = 1)
Bacterial keratitis (n = 10)				
Laboratory confirmed diagnosis (*n* = 8)	None	*n* = 1^c^		*Staphylococcus epidermidis* (*n* = 4) *Staphylococcus aureus* (*n* = 1) *Pseudomonas aeruginosa* (*n* = 1) *Staphylococcus epidermidis* and *Proteus mirabilis* (*n* = 1) *Streptococcus viridans* and *Haemophilus influenza* (*n* = 1)
Clinical diagnosis only (*n* = 2)	None	None		No growth in culture (*n* = 2)

*HIV* human immunodeficiency virus; *HSV-1* herpes simplex virus type 1; *VZV* varicella zoster virus

^a^HSV-1 DNA was detected in combination with *Staphylococcus aureus*

^b^One viral corneal swab was unavailable after transport

^c^Vascular leakage of VZV DNA from extensive corneal neovascularisation most likely resulted in the detection of VZV DNA

Among patients diagnosed with viral keratitis, 15 (52 %) corneal swabs were positive for viral DNA and 14 (48 %) were negative. Positive viral swabs were predominantly obtained from cases with epithelial inflammation (67 %), with dendritic corneal lesions (70 %) as the most common epithelial inflammation, followed by geographic ulcers (20 %). Negative viral swabs were predominantly obtained from cases with subepithelial inflammation (13 of 14, 93 %). Only in one (7 %) case with an epithelial inflammation, presenting with a dendritic lesion typical for HSV-1 epithelial keratitis, could no viral DNA be detected. Notably, subepithelial inflammation was significantly associated with higher median PCR Ct values [median of 38.2 (IQR 37.4–39.1) for subepithelial inflammation vs. 32.6 (IQR 26.7–35.0) for epithelial inflammation, *p* = 0.005]. Furthermore, viral DNA-negative swabs were significantly associated with an increased duration of symptoms [median of 37 (IQR 23–92) days for cases with DNA-negative swabs vs. 14 (IQR 6–18) days for cases with DNA-positive swabs, *p* = 0.003]. A reported history of herpes zoster ophthalmicus (OR = 46.9, 95 % CI 7.4–287.6; *p* < 0.001) was associated with a viral aetiology of keratitis and a trend between viral aetiology and HIV infection (OR = 3.7, 95 % CI 1.1–13.3; *p* = 0.06) was also observed. Among viral keratitis cases, a bacterial superinfection was associated with lower CD4 cell counts [median of 168 (IQR 92–322) cells/mm^3^ for viral keratitis with bacterial superinfection vs. 312 (IQR 212–490) cells/mm^3^ for viral keratitis, *p* < 0.05].

Twenty-one (47 %) keratitis cases were complicated by anterior uveitis, of which 18 (86 %) were viral keratitis. Viral DNA was detected in the corneal swabs of 11 of 18 (61 %) keratouveitis patients: VZV (*n* = 10) and HSV-1 (*n* = 1) (Table 3). Keratouveitis cases were significantly more common among HIV-infected than HIV-uninfected individuals (OR = 16.9, 95 % CI 3.2–89.7; *p* < 0.001), among patients with an intraocular pressure of >21 mmHg than ≤21 mmHg (OR = 5.0, 95 % CI 0.9–27.7; *p* = 0.05) and among viral than bacterial aetiology (OR = 7.6, 95 % CI 1.8–32.7; *p* = 0.005). Also, a trend between lower CD4 cell counts and uveitis was observed among those with HIV infection [median of 226 (IQR 137–332) cells/mm^3^ for keratouveitis vs. 343 (IQR 194–427) cells/mm^3^ for keratitis, *p* = 0.09].

**Table 3:** Factors associated with the development of uveitis in infectious keratitis patients

	Uveitis present (*n* = 21)	Uveitis absent (*n* = 25)	Crude odds ratio (95 % CI)	*p*-Value
Gender				
Female	16 (55)	13 (45)	3.0 (0.8–10.6)	0.13
Male	5 (30)	12 (70)		
Age in years	38 (32–49)	41 (30–62)	na	0.68
Low educational status	10 (53)	9 (47)	1.6 (0.5–5.3)	0.55
Low financial income	18 (86)	18 (72)	2.3 (0.5–10.5)	0.26
HIV infected	19 (91)	9 (36)	16.9 (3.2–89.7)	<0.001
CD4 cell count in cells/mm^3^	226 (137–332)	343 (194–427)	na	0.09
Days between onset of eye complaints and	18 (11–45)	24 (9–39)	na	0.97
Intraocular pressure of >21 mmHg presentation	7 (33)	2 (9)^a^	5.0 (0.9–27.7)	0.05
Diagnosis (clinical and laboratory data combined)				
Viral keratitis (*n* = 15)	7 (47)	8 (53)	7.6 (1.8–32.7)^b^	0.005
Viral and bacterial keratitis (*n* = 14)	11 (79)	3 (24)		
Bacterial keratitis (*n* = 17)	3 (18)	14 (82)		

Data are shown as number (%) or median (interquartile range)

*CI* confidence interval; *p-Value* Pearson Chi-square or Mann–Whitney U test; *na* not applicable; *HIV* human immunodeficiency virus

^a^Three keratitis patients without uveitis had no recorded intraocular pressure

^b^Crude odds ratio and *p*-value were calculated for viral keratitis (including viral and bacterial keratitis) vs. bacterial keratitis

### Clinical outcome of disease

Follow-up after treatment initiation was poor. Thirty-four of 46 (74 %) patients had one or more follow-up visits and the median follow-up time was 7 (IQR 7–28) days. The affected eye was visually impaired at the last follow-up visit after treatment initiation in 17 of 34 (50 %) patients, of which 8 (47 %) were blind. Severe visual impairment and blindness was significantly associated with increased duration of symptoms [median of 36 (IQR 20–112) days for severe visual impairment and blindness vs. 14 (IQR 6–37) days for no severe visual impairment and blindness, *p* = 0.02]. Adjusting for duration of symptoms, no demographic or clinical characteristics were associated with severe visual impairment and blindness (data not shown). Notably, a bacterial superinfection in viral keratitis patients was not associated with poorer outcome after treatment.

## Discussion

This study reports on the clinical and corneal microbial profile of infectious keratitis in patients presenting to the ophthalmology outpatient department of three hospitals in a high HIV prevalence setting in rural South Africa. The data implicate that corneal herpesvirus infections, in part complicated by bacterial superinfection and/or uveitis, are relatively more frequently associated with infectious keratitis in HIV-infected individuals with pronounced visual morbidity. A significant association between HIV infection and keratouveitis was noted, suggesting its potential use as a clinical marker to prompt investigation of the patient’s HIV status.

The observed high frequency of viral aetiology of infectious keratitis in our study has not been described before in sub-Saharan Africa and is higher than observations in similar studies from Australia and China [2, 15]. The major role of herpesviruses might be due to the high HIV prevalence, because a trend was observed between HIV infection and viral keratitis. Our data are in line with observations reporting increased susceptibility of HIV-infected individuals to viral keratitis [7]. We identified VZV as the most prevalent viral cause of infectious keratitis in our study population. This contrasts previous studies from sub-Saharan Africa and high-resource countries that report a predominant role of HSV-1 causing viral keratitis [5, 6, 16]. The important role of VZV in our study might be due to the high HIV prevalence, as HIV-infected individuals are at higher risk for VZV keratitis than for HSV-1 keratitis [7, 17]. Unfortunately, previous studies from sub-Saharan Africa did not report on the patients’ HIV status [5, 6]. Reasons for the observed minor role of HSV-1 in our study remain unclear, as seroprevalence of HSV-1 among HIV-infected ART-naïve individuals in our setting is very high (98 %) [18]. Adenovirus was not detected in our study, which contrasts earlier studies performed in high-resource settings where adenovirus is identified as an important causative pathogen of keratoconjunctivitis [19]. Studies from sub-Saharan Africa are not available, but our results may suggest that geographical factors play a role in the different pathogen distribution observed.

The distribution of bacterial pathogens, largely Gram-positive bacteria, confirms observations in other studies from sub-Saharan Africa [8, 20]. Bacteria were detected in almost half of the viral keratitis cases and bacterial superinfection was associated with lower CD4 cell count. Although limited data are available on the clinical consequences of bacterial superinfections in viral keratitis, bacterial superinfections may worsen the visual outcome of HSV keratitis if appropriate treatment is delayed [21]. In our study, however, poorer visual outcome after treatment was not associated with bacterial superinfection. A fungus (*Candida albicans*) was detected in only one patient diagnosed with bacterial keratitis. This is in sharp contrast to similar studies from Ghana and Tanzania that reported fungi as the causative pathogen in up to 50 % of keratitis cases [22, 23]. This may be due to the exclusion of traumatic keratitis, as trauma is one of the most important risk factors for fungal keratitis, or due to the geographical differences, as fungal keratitis is more likely to occur toward tropical latitudes [2, 3].

PCR analyses of corneal swabs supported the clinical observation of viral keratitis in cases with epithelial inflammation. In cases with subepithelial inflammation, however, the detection of viral DNA was often negative and, if positive, just above the detection limit of the qPCR. This is in line with a study that observed a lower percentage of keratitis cases with positive HSV-1 DNA in patients with subepithelial inflammation compared to patients with epithelial inflammation [24]. Furthermore, increased disease duration was associated with a negative viral swab in cases with a clinical viral keratitis diagnosis.

Anterior uveitis was an important complication of infectious keratitis in our study, in particular in cases of viral aetiology and among HIV-infected individuals. This poses a challenge, as the management of keratouveitis requires specialised treatment. The association between HIV infection and the development of anterior uveitis, combined with the observed trend between immunodeficiency and anterior uveitis, suggest that cell-mediated immunity plays an important role in controlling corneal infection [25, 26]. Also, the presence of anterior uveitis might be used as a pointer of HIV infection that indicates the need for HIV counselling and testing in patients presenting with this condition.

The visual outcome at the last follow-up visit after treatment of infectious keratitis observed in our study was poor and associated with increased duration of symptoms, which confirms observations from a study from Tanzania addressing visual outcome in infectious keratitis [8]. Unfortunately, we did not collect data to determine the reasons for this delay, but both patient- and healthcare system-associated factors may have played a role. A potential contributing factor to the poor visual outcome observed is initial mismanagement at the PHC level, as none of the referred patients from PHC facilities received topical antiviral and/or adequate antibiotic treatment.

A limitation to this study is the small sample size, which might have resulted in an overestimation of the relationships found. Follow-up studies on a larger number of keratitis patients from rural settings with high HIV prevalence are warranted to validate the trends found in this study. Also, we included patients at the outpatient department of hospitals and not at PHC facilities, which may have resulted in some degree of bias towards viral keratitis, as potential bacterial keratitis cases were more likely to be treated successfully at the PHC level. In addition, as we excluded patients with traumatic keratitis and contact lens wearers, it is likely that there is some degree of bias towards viral keratitis, as these are important predisposing factors for microbial keratitis [2]. However, we expect the degree of this bias to be limited, as we excluded only two keratitis cases due to trauma and none for the use of contact lenses.

In conclusion, the results of this study implicate that herpetic keratitis, in part complicated by bacterial superinfection and/or uveitis, is relatively more common among HIV-infected individuals presenting with infectious keratitis in rural South Africa. This warrants an increase of the awareness among healthcare workers in these settings for early clinical signs of herpetic keratitis and prompt initiation of antiviral treatment in these cases to prevent blindness. Moreover, the significant association between HIV infection and keratouveitis warrants examination of the patient’s HIV status.
